# Quasi-Solid-State Electrolyte Induced by Metallic MoS_2_ for Lithium–Sulfur Batteries

**DOI:** 10.1021/acsnano.4c05002

**Published:** 2024-06-04

**Authors:** Zhuangnan Li, Ziwei Jeffrey Yang, James Moloney, Craig P. Yu, Manish Chhowalla

**Affiliations:** † Department of Materials Science and Metallurgy, University of Cambridge, Cambridge CB3 0FS, U.K.; ‡ Yusuf Hamied Department of Chemistry, University of Cambridge, Cambridge CB2 1EW, U.K.; § Cavendish Laboratory, University of Cambridge, Cambridge CB3 0HE, U.K.; ∥ The Faraday Institution, Quad One, Harwell Campus, Didcot OX11 0RA, U.K.

**Keywords:** 2D MoS_2_, Metallic 1T phase, Quasi-solid-state electrolyte, Li−S battery, Cycle life

## Abstract

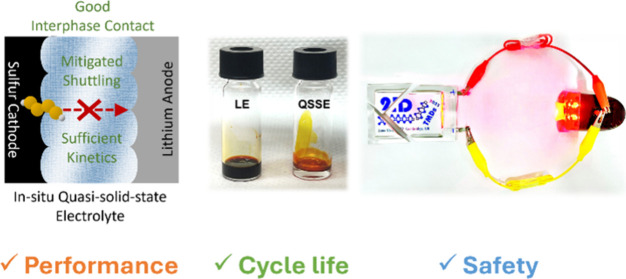

Lithium–sulfur (Li–S) batteries are a promising high-energy-density technology for next-generation energy storage but suffer from an inadequate lifespan. The poor cycle life of Li–S batteries stems from their commonly adopted catholyte-mediated operating mechanism, where the shuttling of dissolved polysulfides results in active material loss on the sulfur cathode and surface corrosion on the lithium anode. Here, we report *in situ* formation of a quasi-solid-state electrolyte (QSSE) on the metallic 1T phase molybdenum disulfide (MoS_2_) host that extends the lifetime of Li–S batteries. We find that the metallic 1T phase MoS_2_ host is able to initiate the ring-opening polymerization of 1,3-dioxolane (DOL), forming an integrated QSSE inside batteries. Nuclear magnetic resonance analysis reveals that the QSSE consists of ∼13% liquid DOL in a solid polymer matrix. The QSSE efficiently mediates sulfur redox reactions through dissolution–conversion chemistry while simultaneously suppressing polysulfide shuttling. Therefore, while ensuring high sulfur utilization, it avoids degradation of both electrodes, as well as the concomitant electrolyte consumption, leading to enhanced cycling stability. Under a practical lean electrolyte condition (electrolyte-to-sulfur ratio = 2 μL mg^–1^), Li–S pouch cell batteries with the QSSE demonstrate a capacity retention of 80.7% after 200 cycles, much superior to conventional liquid electrolyte cells that fail within 70 cycles. The QSSE also enables Li–S pouch cell batteries to operate across a wider temperature range (5 to 45 °C), together with improved safety under mechanical damage.

Lithium–sulfur (Li–S) batteries could be an alternative to lithium-ion energy storage systems due to their high theoretical energy density (∼2600 Wh kg^–1^).^1,2^ Unlike intercalation-type lithium-ion batteries, Li–S batteries operate via the chemical reaction between a sulfur cathode and a lithium metal anode.^3^ The 16-electron transfer process in Li–S batteries usually involves multiple steps. That is, the sulfur cathode dissolves into the ether-based electrolyte to form soluble lithium polysulfides, which undergo liquid phase conversion and precipitate into solid lithium sulfide.^4^ Such dissolution–precipitation chemistry promotes reaction kinetics but causes the shuttling effect.^3−5^ Polysulfide shuttling triggers a series of unfavorable effects in Li–S batteries.^4−7^ For example, it leads to the loss of active material from the sulfur cathode. The reactive polysulfide species can also corrode the lithium metal anode while concomitantly depleting the electrolyte to repair the solid electrolyte interphase (SEI).^5,7,8^ These undesirable processes from the shuttling effect result in premature battery failure, particularly in realistic Li–S pouch cell batteries where limited excessive anodes (low negative-to-positive, N/P, ratio) and electrolyte (low electrolyte-to-sulfur, E/S, ratio) are used.^6^ As a consequence, Li–S pouch cell batteries in the literature rarely run beyond 100 cycles under practical working conditions.^1,7−9^

Strategies have been developed to suppress polysulfide shuttling to improve cycling stability of Li–S batteries.^10−12^ Porous carbons are widely used as sulfur hosts because they provide physical confinement of dissolved polysulfides within the cathode.^13,14^ However, high-porosity carbons require excessive electrolyte, which adds weight but does not contribute to capacity, thus reducing the energy density.^2,6,7,15^ The incorporation of polar sites is commonly used to enhance the chemical adsorption of polysulfides on the cathode.^10,16^ Catalytically active redox mediators are also used to promote electrocatalysis at the cathode–electrolyte interface.^10,11^ While progress has been made on the cathode side, the instability of the anode limits the lifespan of Li–S batteries.^9,17^ In addition to corrosion due to polysulfide shuttling, other challenges related to lithium metal anodes persist. These include dendrite formation, dead lithium, unstable SEI, and structural pulverization.^18,19^ Solid-state electrolytes (SSEs) can address the challenges of a pure lithium anode.^19−21^ However, SSEs generally possess low ionic conductivity and large interfacial resistance compared to liquid electrolytes (LEs).^20^ More importantly for Li–S batteries, the compatibility of SSEs with sulfur cathodes is poor, which reduces sulfur utilization.^19^ This is because the insulating nature of both sulfur and lithium sulfide results in a high solid to solid conversion kinetic barrier.^3,20,21^ For this reason, Li–S chemistry relies on the catholyte-mediated reaction mechanisms.^2,6,7,10^ Therefore, while an enhancement in cycle life might be feasible, high energy density Li–S batteries with SSEs are challenging.

In this work, we report the realization of considerably stable Li–S batteries using a quasi-solid-state electrolyte (QSSE) induced by a metallic 1T phase molybdenum disulfide (1T MoS_2_) host. The QSSE is formed *in situ* and thereby well integrated into the battery, addressing the interfacial resistance problem resulting from poor contact. In contrast to SSEs, such a QSSE contains a small fraction of liquid solvents, which ensures adequate Li^+^ ion transport. More importantly, the liquid regions enable the dissolution-based polysulfide reaction pathways, rendering faster redox kinetics relative to the solid–solid conversion route. Furthermore, while achieving facile charge transfer in solution, the high viscosity of the QSSE suppresses polysulfide shuttling and thus mitigates corrosion of lithium metal anodes. Li–S pouch cells using a QSSE deliver comparable specific capacity to those with conventional electrolytes, but exhibit over 3-fold longer cycle life, retaining >80% of the initial capacity after 200 cycles. The QSSE-based Li–S batteries also show stable operation over an extended temperature range and superior safety under mechanical damage, making them potentially feasible for practical applications.

## Results and Discussion

Synthesis of the QSSE is underpinned by the ring-opening polymerization of 1,3-dioxolane (DOL). This reaction has been studied for over 60 years and is typically initiated by a Lewis acid.^22,23^ However, the initiators usually remain as impurities in the electrolyte or require further purification steps during battery manufacturing. Unlike conventional routes, we use the sulfur host material—1T MoS_2_—to initiate the polymerization of liquid DOL precursor inside the assembled cells. This *in situ* induced polymerization is facile, high in purity, and produces an excellent interface between the electrode and electrolyte (Figures S1 and S2), leading to low interfacial resistance.

**Figure 1:**
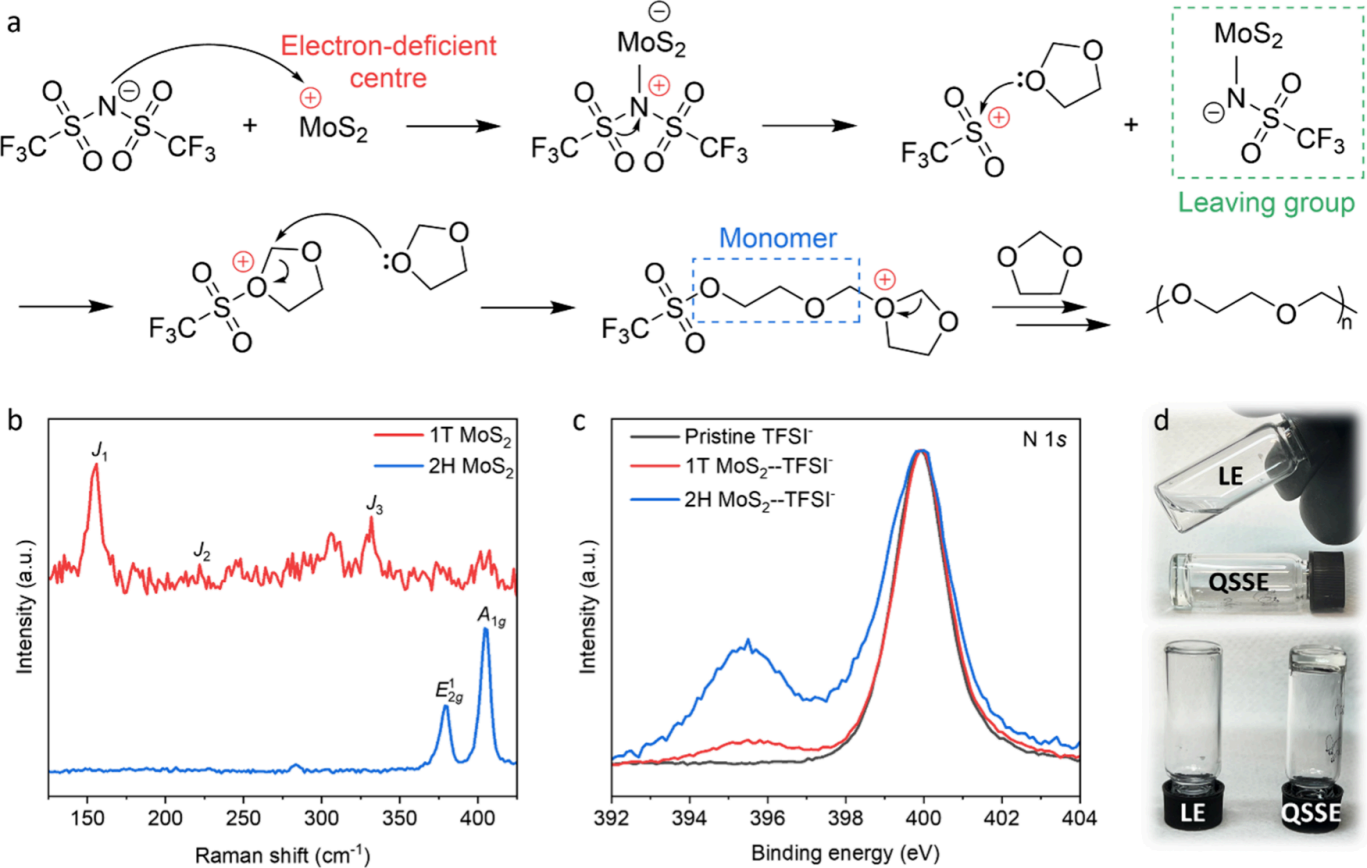
Preparation of the QSSE. (a) Reaction mechanism illustrating the ring-opening polymerization initiated by MoS_2_-based cathodes. It can be seen that the interaction between the TFSI^–^ anion and MoS_2_ generates an electron-deficient center, which is attacked by the lone-pair electron in DOL, triggering the ring-opening reaction. (b) Raman spectra of 1T MoS_2_ and 2H MoS_2_. The *A*_1*g*_ and *E*_2*g*_^1^ peaks are the feature of the semiconducting 2H phase, while the *J*-series peaks indicate the presence of the metallic 1T phase. (c) High-resolution XPS spectra of N 1*s*, revealing a weaker interaction (peak at ∼395.5 eV) between 1T MoS_2_ and TFSI^–^ anions compared to 2H MoS_2_. (d) Photographs of the original LE, and QSSE induced by metallic 1T MoS_2_, showing the initially fluid LE transforms into the immovable and transparent QSSE upon polymerization.

Figure 1a illustrates a cationic ring-opening polymerization reaction initiated by MoS_2_-based cathodes. Specifically, the Lewis acidic Mo site in MoS_2_ interacts with the bis(trifluoromethanesulfonyl)imide (TFSI^–^) anion in the electrolyte, creating an electron-deficient N center. Subsequently, the electron of the adjacent S atom is transferred to the electropositive N site due to the relatively high electronegativity of the N atom. This in turn generates a sulfonyl leaving group and a residual electron-deficient S center. The electropositive S site is then attacked by the lone-pair electron of the O atom in DOL, forming an oxonium ion that initiates the ring-opening polymerization of DOL monomers. As the polymer chains grow, the electrolyte transforms from its original liquid state to an immovable solid. It is noteworthy that the ring-opening reaction is primarily associated with ring strain and therefore will be greatly affected by the composition of the electrolyte. For example, the ring-opening polymerization of DOL is inhibited in electrolyte-containing lithium nitrate (LiNO_3_) due to the strong coordination between DOL and LiNO_3_ (Figure S3).^24,25^ In addition, we attribute the activation of polymerization to the Lewis acidic site produced by the interaction between MoS_2_ and TFSI^–^, rather than to the Mo atom in MoS_2_. This is because, although MoS_2_ is capable of inducing the ring-opening polymerization, the presence of TFSI^–^ anions in the electrolyte renders higher Lewis acidity and consequently accelerates such a reaction (Figure S3). A similar mechanism has been proposed previously, in which the reduction in electrochemical performance was ascribed to a gel layer covering the surface of MoS_2_.^26^ Here we show that the gelation process can be controlled by tuning the intrinsic properties of MoS_2_, leading to a well-integrated QSSE that substantially enhances the cycling stability of Li–S batteries.

**Figure 2:**
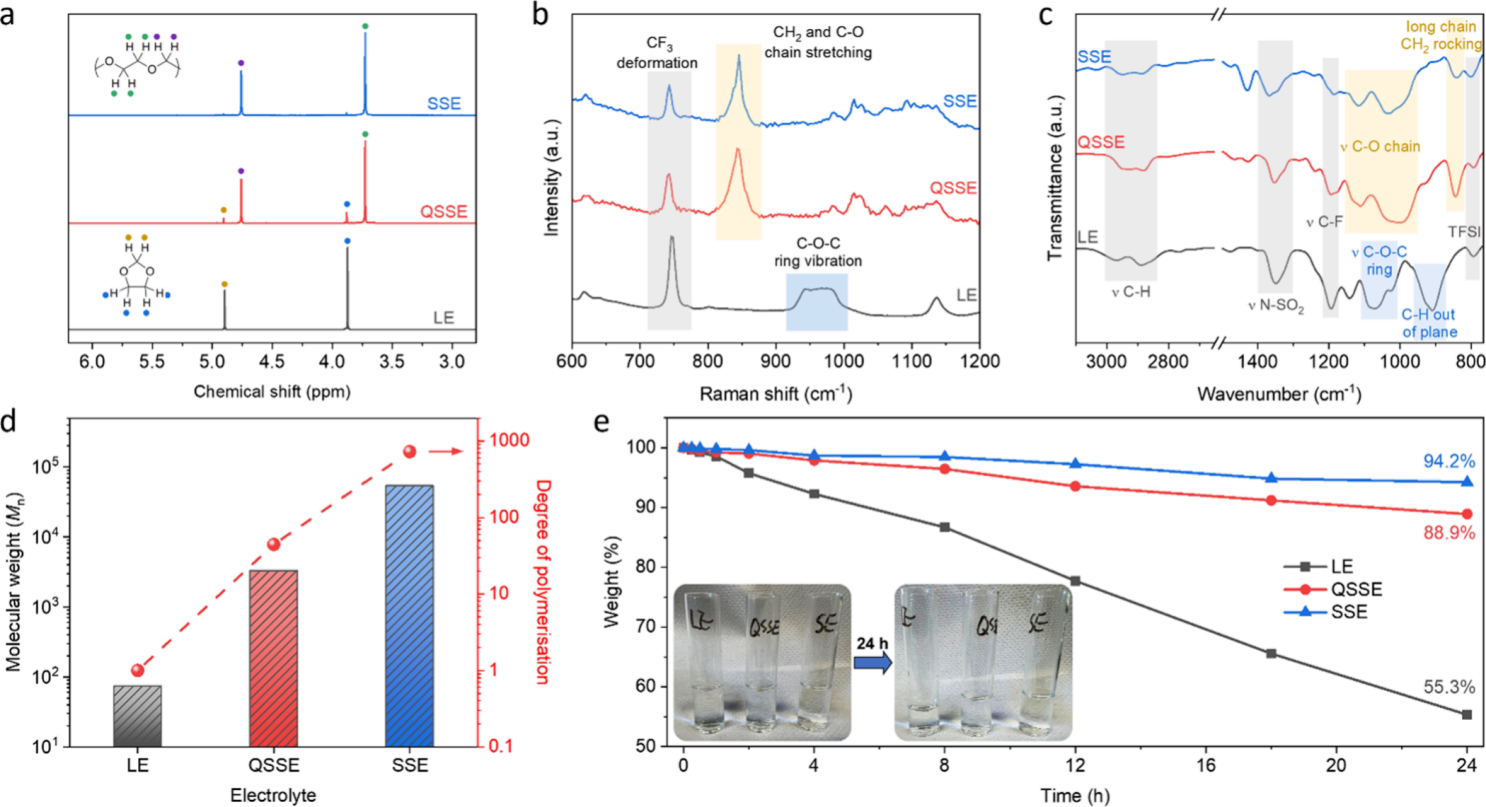
Structural characterization of different electrolytes. (a) ^1^H NMR spectra with the relative hydrogens labeled on the molecular structures and chemical shifts, indicating that the QSSE incorporates a portion of the unpolymerized liquid into its polymer framework. (b, c) Raman (b) and FTIR (c) spectra with characteristics of liquid monomer, solid polymer, and electrolyte salt shaded in blue, yellow, and gray colors, accordingly. It can be seen that the QSSE exhibits solid-like properties. (d) Number-average molecular weight of different electrolytes and their corresponding degree of polymerization, showing that the QSSE possesses a 10-fold lower polymerization degree compared with the SSE. (e) Weight as a function of time for different electrolytes leaving in an uncapped vial (inset), suggesting superior resistance of the QSSE against volatilization loss.

MoS_2_ is layered and exists in the semiconducting 2H phase (2H MoS_2_).^27,28^ As described in Figure 1, 2H MoS_2_ with strong Lewis acidity can readily initiate the ring-opening polymerization in typical Li–S LEs that contain DOL and TFSI^–^, yielding a polymer-based SSE. Chemical treatment of semiconducting 2H MoS_2_ by organolithium enables its transformation into the metallic 1T phase (Figure 1b and Experimental Methods), during which an electron is donated to the MoS_2_ structure from the organic group.^28^ The extra electron partially neutralizes the Lewis acidity of 1T MoS_2_, and thus weakens its interaction with TFSI^–^ anions compared to 2H MoS_2_ (Figure 1c). Consequently, the polymerization process initiated by 1T MoS_2_ terminates at a lower reaction degree than that with 2H MoS_2_, producing a transparent QSSE (Figure 1d), a polymer framework incorporating a portion of unpolymerized liquid. The fraction of the unreacted liquid can be estimated with nuclear magnetic resonance (NMR). Specifically, in ^1^H NMR spectra (Figure 2a), the LE shows chemical shifts at 3.87 and 4.90 ppm, representing the H atoms on the DOL monomer ring. After the ring-opening reaction, new peaks corresponding to the H sites on the polymer chain appear at 3.73 and 4.76 ppm for both the QSSE and SSE. It can be seen that partial liquid DOL remains in the QSSE, while it is negligible in the SSE. Integrating the peak area reveals that the unpolymerized original DOL fraction in QSSE is ∼13%. These structural changes have been further confirmed by Raman spectroscopy (Figure 2b) and Fourier-transform infrared spectroscopy (FTIR) (Figure 2c), where both the QSSE and SSE exhibit C–O chain stretching in lieu of the C–O–C ring vibration observed in LE.

In addition to the unpolymerized liquid fraction, the solid products in the QSSE possess a lower degree of polymerization compared to those in the SSE. More specifically, polymers after purification were investigated by gel permeation chromatography (Figure 2d). The number-average molecular weight (*M*_n_) of the QSSE was determined to be ∼3300 g mol^–1^, which is more than an order of magnitude smaller than that of the SSE (∼53700 g mol^–1^), suggesting over 10-fold lower polymerization degree (*M*_n_/*M*_0_, *M*_0_ is the weight of a monomer unit). Furthermore, by formation of the polymer framework, the QSSE greatly enhances its resistance against volatilization. That is, unlike the LE, which has a weight loss of ∼45% within 24 h under ambient conditions (25 °C, atmospheric pressure), the QSSE retains around 89% of its initial weight (Figure 2e).

**Figure 3:**
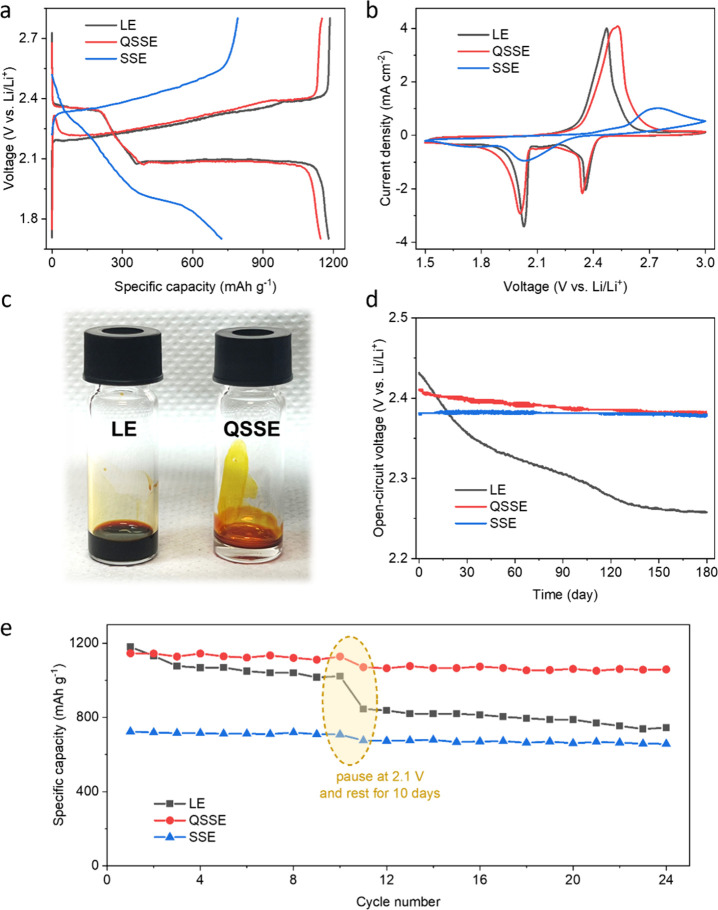
The role of the QSSE on sulfur-based cathodes. (a) Galvanostatic charge–discharge curves of Li–S coin cells with different electrolytes at a current density of 0.1 C. (b) Cyclic voltammetry curves of Li–S coin cells with different electrolytes at a scan rate of 0.1 mV s^–1^. It can be seen in both GCD and CV curves that the QSSE enables the sulfur-based cathode to operate with the catholyte-mediated mechanism. (c) Permeation behavior of Li_2_S_6_ in LE and QSSE for 10 h, showing restricted migration of polysulfides in the QSSE. (d) Open-circuit voltage of assembled Li–S coin cells with different electrolytes, indicating low self-discharge and a long shelf life of QSSE-based cells. (e) Cycling stability of Li–S coin cells with different electrolytes. All cells were paused at 2.1 V of their 11th discharge process and rested for 10 days before resuming.

**Figure 4:**
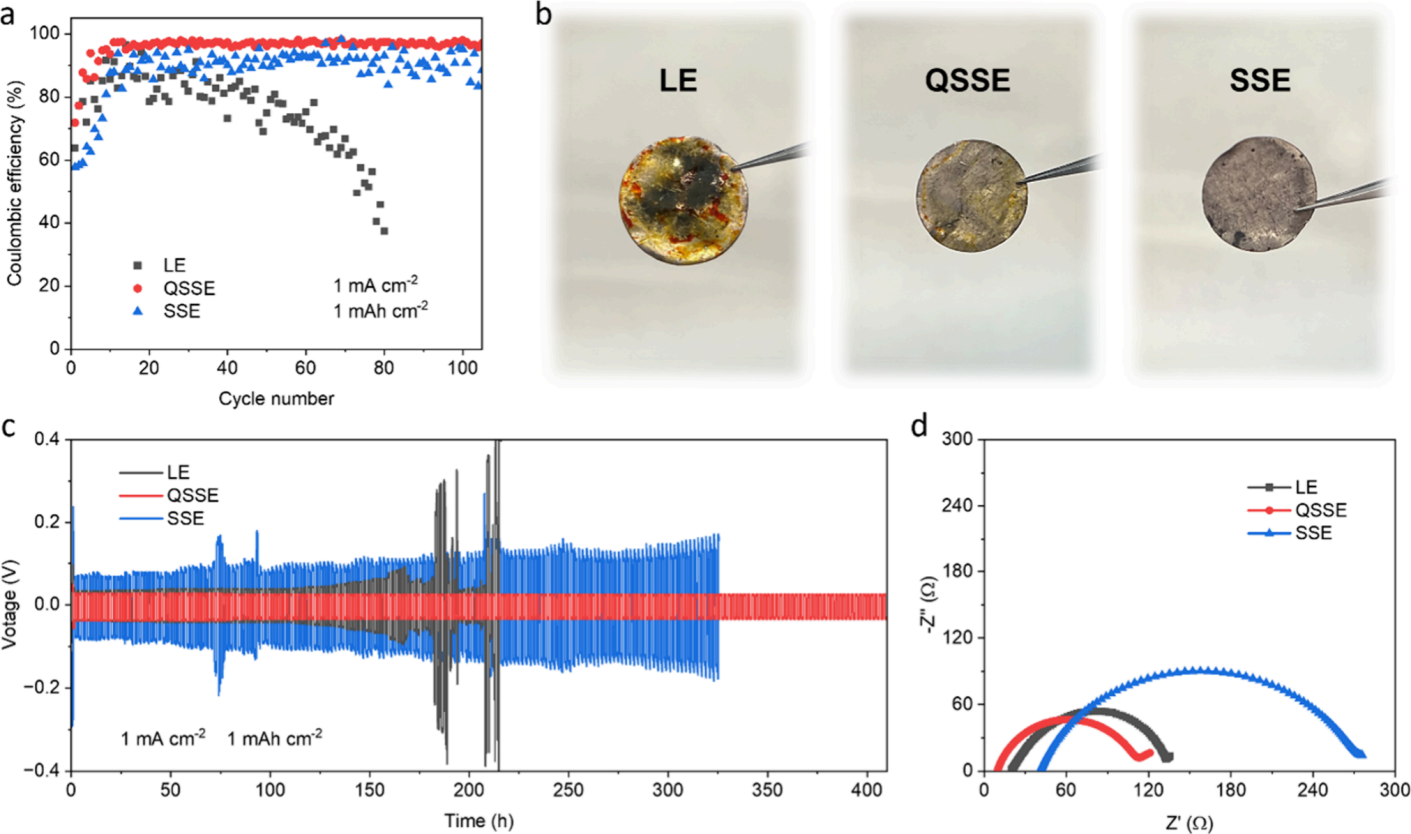
The role of the QSSE on lithium metal anodes. (a) Coulombic efficiency of the Li stripping and plating process in asymmetric Li||Cu cells with different electrolytes. (b) Photographs of the lithium metal anodes after adding Li_2_S_6_ to the cells with different electrolytes, indicating greatly mitigated anode corrosion by the QSSE and SSE. (c) Long-term cycling performance of symmetric Li cells with different electrolytes. (d) Nyquist plots of symmetric Li cells with different electrolytes after 200 cycles, showing the smallest resistance of QSSE-based cells.

The electrochemical properties of the QSSE were investigated by understanding its role in Li–S chemistry on the cathode (Figure 3) and anode (Figure 4). To evaluate the effect on sulfur-based cathodes, we assembled a series of coin cells with different electrolytes (Experimental Methods). In these cells, QSSEs and SSEs were formed *in situ* by employing 1T MoS_2_ and 2H MoS_2_ initiators as the sulfur host material, respectively. For LE, to maintain its liquid state, LiNO_3_ was introduced as an additive to inhibit ring-opening polymerization. In addition, we kept both the electrolyte (E/S ratio = 15 μL mg^–1^) and the anode (400 μm thickness) in excess for testing the limits of the cathode performance.

Galvanostatic charge–discharge (GCD) curves of the Li–S cells with the QSSE exhibit two discharge plateaus at 2.4 and 2.1 V (Figure 3a), similar to those with the LE, suggesting a typical catholyte-mediated mechanism. Considering that the dissolution of polysulfides in the electrolyte is primarily ascribed to the 1,2-dimethoxyethane (DME) solvent,^29^ the presence of two characteristic plateaus indicates that the cathode surface is still accessible to DME in the QSSE. In comparison, plateaus are less obvious in GCD curves of cells with the SSE, revealing that DME cannot access the cathode in the SSE, and thus the cells have to undergo a solid–solid sulfur conversion reaction. We attribute this difference to the retention of unpolymerized DOL in the QSSE, which is known to be highly compatible with DME.^30^ As a consequence, sulfur cathodes in the QSSE through a dissolution-based stepwise reaction pathway deliver a specific capacity of 1146 mAh g^–1^ at 0.1C, much higher than the cathodes in the SSE (723 mAh g^–1^) and even comparable to those in the LE (1180 mAh g^–1^). Cyclic voltammetry (CV) results of the QSSE-based cells also exhibit two representative cathodic peaks and one anodic peak (Figure 3b), consistent with the GCD profiles. Moreover, sulfur redox kinetics studied by Li_2_S deposition and Li_2_S_6_ conversion further confirm that the QSSE enables similar electrochemical behavior to that of the LE (Figure S4).

Dissolution of polysulfides is responsible for the high specific capacity of cathodes, but it is generally the root of the shuttling effect that results in poor cycling stability.^5^ In our QSSE, while allowing the catholyte-mediated Li–S chemistry, the polymer framework prevents polysulfides from penetrating through the electrolyte, and thereby adequately suppresses their shuttling (Figure 3c). In addition, the confinement of polysulfides to the cathode side ensures the low self-discharge rate and long shelf life of QSSE-based Li–S batteries. It can be seen in Figure 3d that the open-circuit voltage (OCV) of freshly assembled cells with LE drops by 0.08 V (from 2.43 V to 2.35 V) after resting for 30 days and further decreases to below 2.3 V over 100 days. In this regard, QSSE-based cells show an OCV drop of only 0.01 V (from 2.41 V to 2.4 V) after 30 days and remains above 2.38 V after 180 days. The self-discharge current in the QSSE is also consistently lower across all the OCVs compared to the LE (Figure S5). Such a reduction in self-discharge behavior and extension of shelf life with QSSE are crucial for practical application of Li–S batteries. This is because Li–S batteries generally possess poor static electrochemical stability, suffering from severe self-discharge with a capacity decay of over 50% in a month,^31^ which has hindered their real-world feasibility. To further evaluate self-discharge, more realistic measurements were carried out by pausing discharged cells at 2.1 V, where the polysulfides are most concentrated. After 10 days, the resumed Li–S cells with the QSSE demonstrate a capacity loss of 4% (comparable to commercial Li-ion batteries: <3% per month) in subsequent cycles, much smaller than the ∼17% loss with the LE.

To investigate the role of the QSSE on the anode side, we assembled asymmetric Li||Cu cells and measured their galvanostatic polarization behavior at 1 mA cm^–2^. Figure 4a shows that the Coulombic efficiency of cells with LE fades rapidly after 50 cycles of Li stripping and plating. We ascribe this to the instability of the SEI formed from the LE, which fractures in each cycle and is repaired by continuously consuming the electrolyte. In contrast, cells with the QSSE and SSE are stable for more than 100 cycles. A comparably low and fluctuating Coulombic efficiency is observed in the SSE due to its relatively poor ionic conductivity. Moreover, the SEI formed in polymer-based electrolytes (QSSE and SSE) also provides better protection to the lithium metal anode from parasitic reactions—for example, the shuttling effect—than that in the conventional LE. This enhanced protection is demonstrated by the lower degree of anode corrosion when Li_2_S_6_ was added to the cells (Figure 4b). The long-term reversibility of Li stripping and plating in different electrolytes was further evaluated in symmetric Li cells. As seen in Figure 4c, the polarization in the SSE is consistently higher than that in the LE and QSSE during the initial 150 cycles. This is associated with the lower ionic conductivity, in agreement with results from asymmetric Li||Cu cells. It is noteworthy that the polarization in the LE and QSSE are similar initially, but a gradual increase in overpotential is observed in LE-based cells with cycling. The higher overpotential means that a larger driving force is required to strip and plate Li in each cycle, which is unfavorable for Li deposition. Such a process is generally believed to result in uncontrolled lithium growth, accumulation of dead lithium, and ultimately internal short circuits.^17,18,32^ In comparison, cells using the QSSE operate stably for more than 400 h, together with the smallest resistance after cycling (Figure 4d), suggesting better compatibility of the QSSE with the lithium metal anodes.

**Figure 5:**
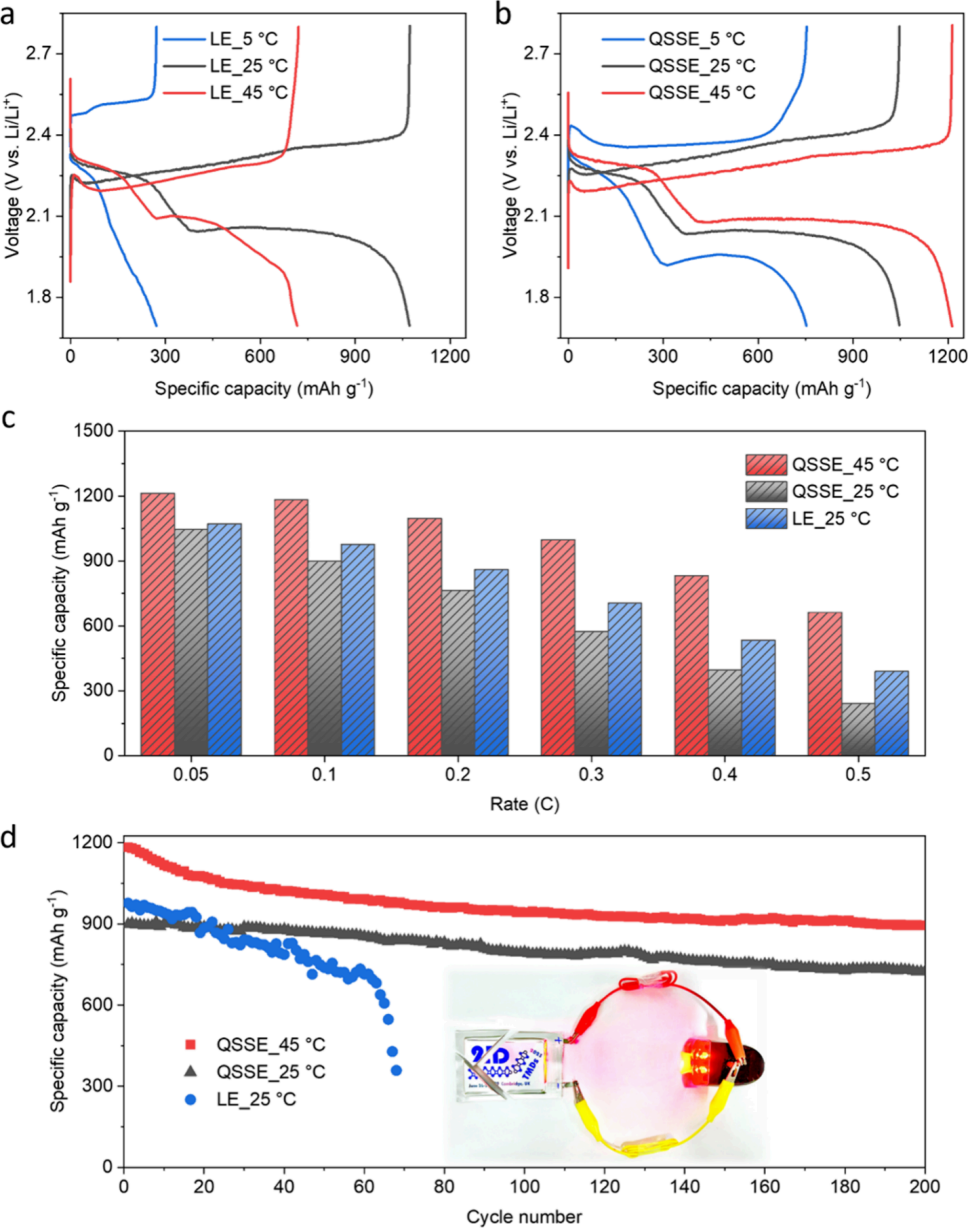
Fabrication of QSSE-based Li–S pouch cells. (a, b) Galvanostatic charge–discharge curves of Li–S pouch cells with the LE (a) and QSSE (b) at a current density of 0.05 C under various temperatures. It can be seen that QSSE-based cells operate stably at these temperatures. (c) Specific capacities of Li–S pouch cells with the LE (under 25 °C) and QSSE (under 25 and 45 °C) at different current densities. (d) Cycling stability of Li–S pouch cells with the LE (under 25 °C) and QSSE (under 25 and 45 °C) at a current density of 0.1 C, exhibiting >80% capacity retention of QSSE-based cells over 200 cycles. The inset photograph shows light-emitting diodes powered by a QSSE-based Li–S pouch cell under mechanical damage.

The above results provide structural information about the QSSE (Figure 2), along with its role on the cathode (Figure 3) and anode (Figure 4) in Li–S chemistry. Building on these findings, we fabricated pouch cell level Li–S batteries with the *in situ* formed QSSE (Figure 5).

As a high-energy-density energy storage technology, Li–S batteries have been broadly considered as a suitable power supply for applications such as drones and low-orbit satellites.^2,33^ To this end, a wide operating temperature range is desirable for Li–S batteries. However, this is intrinsically challenging with conventional LEs due to the severe shuttling effect at high temperatures and sluggish reaction kinetics at low temperatures.^30,34,35^ We therefore sought to extend the working temperature range of Li–S batteries using the QSSE. It can be seen in the GCD curves that both Li–S pouch cell batteries with the LE and QSSE exhibit a specific capacity of ∼1050 mAh g^–1^ at room temperature (25 °C) (Figure 5a and b). At 45 °C, although LE-based pouch cells show enhanced reaction kinetics (smaller polarization voltage gap between charge and discharge curves), their specific capacity (716 mAh g^–1^) drops significantly. These results indicate the exacerbated shuttling of polysulfides in the LE at high temperature, as also indicated by the poorly maintained second discharge plateau (Figures 5a and S6). In contrast, QSSE-based pouch cells deliver a higher specific capacity of 1213 mAh g^–1^ as a result of enhanced kinetics at a high temperature (Figure 5b). Furthermore, at 5 °C, where LE-based cells can barely operate, the Li–S pouch cell batteries using the QSSE retain a considerable specific capacity of 753 mAh g^–1^, suggesting good low-temperature adaptability. The QSSE-based pouch cells also demonstrate superior rate capability, achieving 55% capacity retention at 0.5 C, which is ∼20% higher than that of cells with the LE (Figure 5c).

**Figure 6:**
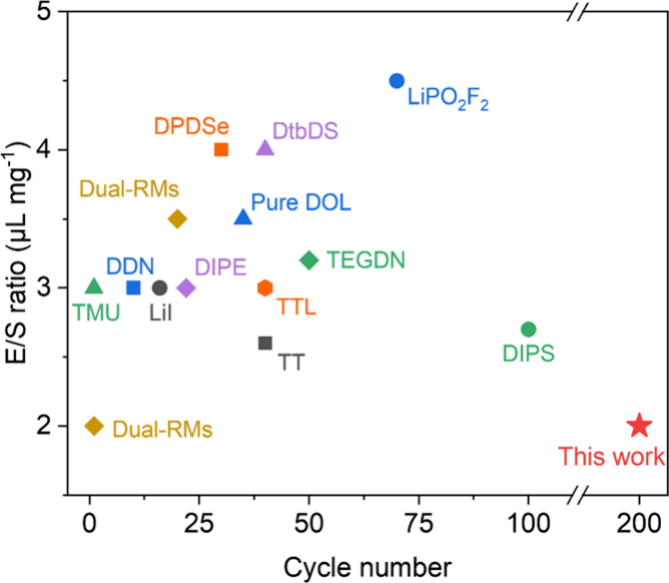
Cycle life and E/S ratio comparison of QSSE-based Li–S batteries with reported pouch cells using different electrolytes.^26,38−47^

To date, the cycle life of Li–S batteries is far from being suitable for practical applications. Most pouch cells with LEs reported in the literature usually fail within 100 cycles.^8,9,36,37^ The failure mechanisms have been traced to a combination of cathode polysulfide shuttling, electrolyte depletion, and anode corrosion.^8,36^ More specifically, in LE-based Li–S batteries with high energy density (for example, >350 Wh kg^–1^ at the pouch cell level), the depletion of electrolyte is the limiting factor for cycling stability.^9,37^ Our previous study demonstrated that by supplying adequate electrolyte (E/S ratio = 2.4 μL mg^–1^) in Li–S pouch cells with metallic MoS_2_ cathodes, it is possible to retain ∼85% of capacity after 200 cycles.^15^ However, in pursuit of higher energy density by further reducing electrolyte volume (E/S ratio = 2 μL mg^–1^), cycling stability of the cells with the LE drops dramatically, failing after <70 cycles (Figure 5d). In this respect, QSSE-based Li–S pouch cell batteries operate beyond 200 cycles while still retaining 80.7% of their original capacity (Figure 5d), which is over 3-fold the lifetime of conventional LE-based cells under identical conditions. Such a cycle life at low E/S ratios is also among the highest reported thus far for Li–S batteries with different electrolytes (Figure 6).^26,38−47^ Postcycling analysis of these QSSE-based cells reveals that anode degradation is the primary mechanism responsible for performance decay (Figure S7). In addition, the safety of batteries is important. As seen in the inset of Figure 5d, our Li–S pouch cell batteries using the QSSE are capable of safe operation even under mechanical damage. The above improvements in working temperature range, cycle life, and safety demonstrate the promise of implementing QSSEs in next-generation Li–S batteries.

## Conclusions

In summary, we report the use of QSSE induced by metallic 1T MoS_2_ hosts to extend the cycle life of Li–S batteries. The QSSE is formed *in situ* within the assembled cells and thus produces good interfaces on both electrodes. In addition, the moderate Lewis acidity of 1T MoS_2_ enables the resultant QSSE to incorporate a portion of the unpolymerized liquid into its polymer framework. Therefore, while suppressing the shuttling of polysulfides, the QSSE allows the Li–S cells to proceed with the catholyte-mediated reaction route, which largely promotes the sulfur redox chemistry. On the other hand, this QSSE integrated into cells in lieu of conventional LEs mitigates anode corrosion and electrolyte depletion problems common to all Li–S batteries. These attributes collectively improve the cycling stability of Li–S batteries without sacrificing performance. The fabricated pouch cell level Li–S batteries using QSSE can retain their initial capacity in excess of 80% after 200 cycles, more than a 3-fold increase in cycle life compared to state-of-the-art cells. Furthermore, our QSSE-based pouch cells show wide temperature adaptability and good safety, demonstrating potential for applications.

## Experimental Methods

### Preparation of MoS_2_-Based Sulfur Hosts and Their Sulfur Composites for Cathodes

2H MoS_2_ powder (Alfa Aesar) was used as purchased without further modification. 1T MoS_2_ was synthesized by chemical exfoliation of bulk 2H MoS_2_ with organolithium as reported previously.^15,28,48^ Briefly, bulk 2H MoS_2_ powder (0.3 g) was first immersed in hexane (15 mL; Sigma-Aldrich), followed by adding *n*-butyllithium solution (1.6 M in hexane, 3 mL; Sigma-Aldrich) and refluxing for 2 days under argon protection. The product was then washed with hexane (50 mL) 3 times and dispersed in deionized water (1 mg mL^–1^) with the aid of ultrasonication (20 min). After centrifugation to remove the unreacted parts and residues, the resultant powder was freeze-dried to yield 1T MoS_2_.

Sulfur composites were prepared by mixing MoS_2_ host material (40 mg) and sulfur powder (100 mg; Alfa Aesar) through a ball milling process. The mixture was next ground with poly(vinylidene fluoride) binder (MTI Corporation) at a mass ratio of 9:1 in *N*-methyl-2-pyrrolidone (Sigma-Aldrich) to form a homogeneous slurry. Note that for the slurry of 2H MoS_2_, an additional 10 wt % Super P carbon (MTI Corporation) was added to ensure sufficient conductivity (that is, sulfur composite, carbon, binder at a 8:1:1 mass ratio). The slurry was then coated onto Al foils (MTI Corporation) using a doctor blade and dried at 60 °C for 24 h. The areal sulfur loadings were 2.5 and 7.5 mg cm^–2^ for coin cells and pouch cells, respectively.

### Preparation of Electrolytes and Polysulfide Solutions

All electrolytes and polysulfide solutions were prepared in an argon-filled glovebox. A LiTFSI slat (1.0 M; Sigma-Aldrich) was first dissolved in mixed DOL and DME (1:1 by volume; Sigma-Aldrich) solvents to produce a liquid precursor. The LE was prepared by dissolving LiNO_3_ (0.2 M; Sigma-Aldrich) in the precursor. The QSSE and SSE were formed *in situ* by dropping the liquid precursor onto 1T MoS_2_- and 2H MoS_2_-based cathodes, respectively, during cell assembly. Note that the QSSE and SSE without integration into cells can also be obtained by adding corresponding MoS_2_ powders (0.5 mg) to the liquid precursor (2 mL). Polysulfide solutions were prepared by mixing sulfur and lithium sulfide (Alfa Aesar) powders in stoichiometric proportion in the precursor, followed by stirring overnight at 50 °C.

### Materials Characterization

Morphological and structural information on materials were detected by scanning electron microscopy (FEI Magellan 400), X-ray photoelectron spectroscopy (ThermoFisher Scientific using an Al Kα source), NMR spectroscopy (Bruker 400 MHz Avance III HD using chloroform-*d* as a deuterated solvent), Raman spectroscopy (Renishaw InVia using a 514 nm laser beam), FTIR spectroscopy (Bruker Tensor 27 using an ATR mode), and gel permeation chromatography (Agilent 1260 Infinity II using tetrahydrofuran as the mobile phase).

### Electrochemical Characterization

Electrochemical performance of electrolytes was characterized in coin cells (CR2032) and pouch cells (6 cm × 4.5 cm in dimension). All of the coin cells were fabricated in an argon-filled glovebox. Li–S coin cells were assembled with the sulfur-based cathode, the lithium foil anode, Celgard separator, and the electrolyte (E/S ratio = 15 μL mg^–1^). Asymmetric Li||Cu and symmetric Li coin cells were assembled with similar components but with changing the electrodes to lithium foil versus Cu foil and two pieces of lithium foil, respectively. Symmetric Li_2_S_6_ coin cells were also assembled with similar components but with changing the electrodes and electrolyte to two identical hydraulically pelletized MoS_2_ (25 MPa) and 0.2 M Li_2_S_6_ solutions (50 μL), respectively. Pouch cells were fabricated in a dry room (relative humidity <0.1%) with the sulfur-based cathode (Al current collector), the lithium foil anode (Cu current collector), Celgard separator, and the electrolyte (E/S ratio = 2 μL mg^–1^). The Al and Ni tabs were welded together with the cathodes and anodes, respectively. The entire cell core was encapsulated in the Al-laminated films.

GCD tests were carried out on a battery cycler (LANHE CT3002A) in the voltage range of 1.7 to 2.8 V at various C rates (1 C = 1672 mAh g^–1^). An oven and a fridge were coupled to the battery cycler to control the temperature during GCD measurements. CV tests were conducted with an electrochemical workstation (BioLogic VSP-300) from 1.5 to 3.0 V at various scan rates. Li_2_S deposition tests were performed by galvanostatically discharging fresh coin cells to 2.06 V at a current density of 0.05 C, followed by potentiostatically discharging at 2.05 V until the current was below 10^–2^ mA. Li_2_S_6_ conversion tests were studied in symmetric Li_2_S_6_ cells by CV scans from −0.5 to 0.5 V at a rate of 50 mV s^–1^. OCV of freshly assembled cells before measurements was recorded for 180 days to monitor the self-discharge behavior. Self-discharge current at various OCVs was examined after the cells reached voltage equilibrium during the relaxation period, using the previously reported method.^49^ The cycling stability was evaluated by performing continuous GCD cycles. The Li stripping and plating tests were investigated in both asymmetric Li||Cu (stripping cutoff voltage = 1 V) and symmetric Li cells at various current densities. Electrochemical impedance spectroscopy was measured at open circuit under a sinusoidal signal over the frequency range from 100 kHz to 100 mHz with an amplitude of 10 mV.

## Supporting Information Available

Additional data of schematic of Li–S batteries with different electrolytes, SEM images of cathodes, photographs of the QSSE formation process with different precursors, electrochemical kinetics study of different electrolytes, self-discharge current of cells with different electrolytes at various temperatures, and battery degradation mechanism study (PDF)
