# Supplementary material for: Quasi-Solid-State Electrolyte Induced by Metallic MoS2 for Lithium–Sulfur Batteries
Source: ACS Nano. 2024 Jun 4;18(24):16041–50. doi: 10.1021/acsnano.4c05002 (PMC11191740; doi:10.1021/acsnano.4c05002)
Supplement: Supplementary file 1 — nn4c05002_si_001.pdf [file nn4c05002_si_001.pdf]

Supporting information

Quasi-solid-state electrolyte induced by metallic MoS_2 for lithium-sulfur batteries

Zhuangnan Li[1], *Ziwei Jeffrey Yang*[1], *James Moloney*[1], *Craig P. Yu*[2,3] *and Manish Chhowalla*[1,4]*

[1]Department of Materials Science and Metallurgy, University of Cambridge, Cambridge CB3 0FS, UK

[2]Yusuf Hamied Department of Chemistry, University of Cambridge, Cambridge CB2 1EW, UK

[3]Cavendish Laboratory, University of Cambridge, Cambridge CB3 0HE, UK

[4]The Faraday Institution, Quad One, Harwell Campus, Didcot OX11 0RA, UK

*email: mc209@cam.ac.uk

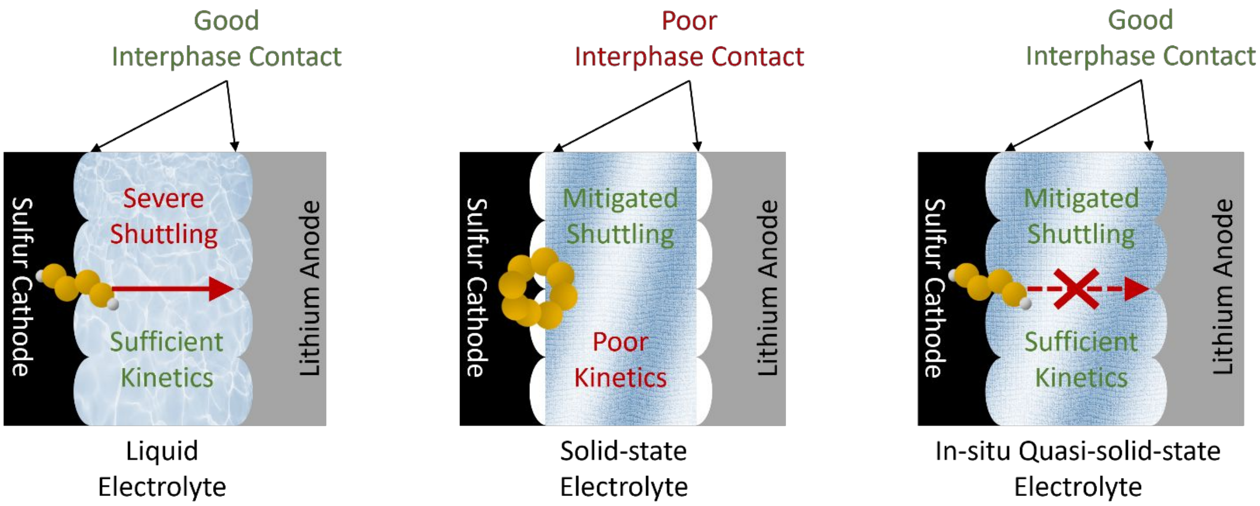

Figure S1. Schematic of Li-S batteries with different electrolytes, illustrating that the *in-situ* formed QSSE in Li-S batteries ensures good contact with electrodes, mitigates polysulfides shuttling, and promotes reaction kinetics.

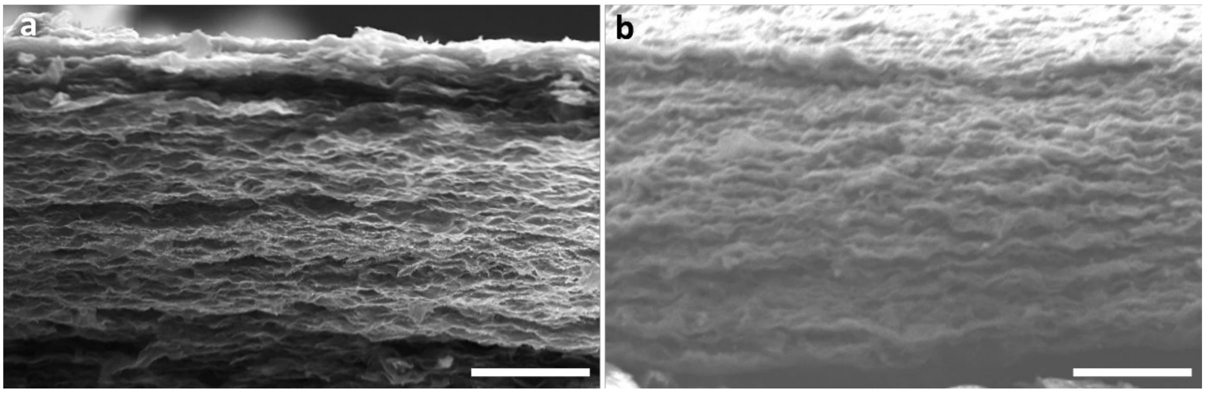

Figure S2. SEM images showing the morphology of 1T MoS_2-based cathodes before **(a)** and after **(b)** QSSE integration. Scale bars, 1 µm.

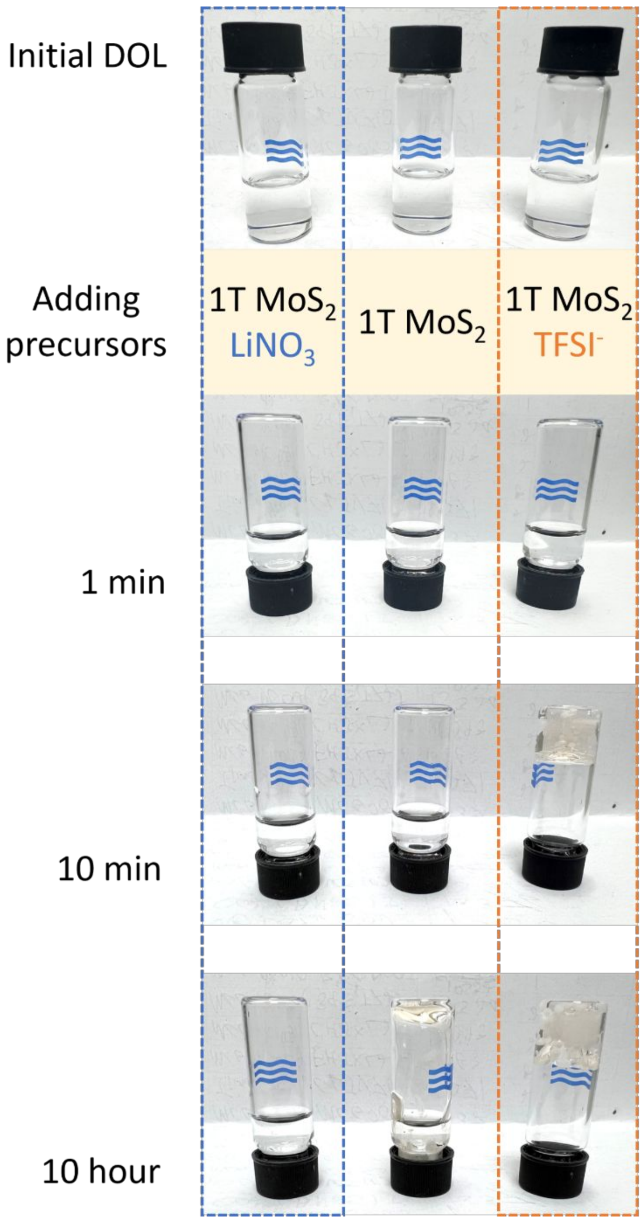

Figure S3. Photographs of QSSE formation process with different precursors. The results indicate that 1T MoS_2 alone is capable of forming QSSE though at a relatively slow reaction rate. This formation reaction of QSSE can be accelerated by $TFSI^-$ anions and inhibited in the presence of $LiNO_3$.

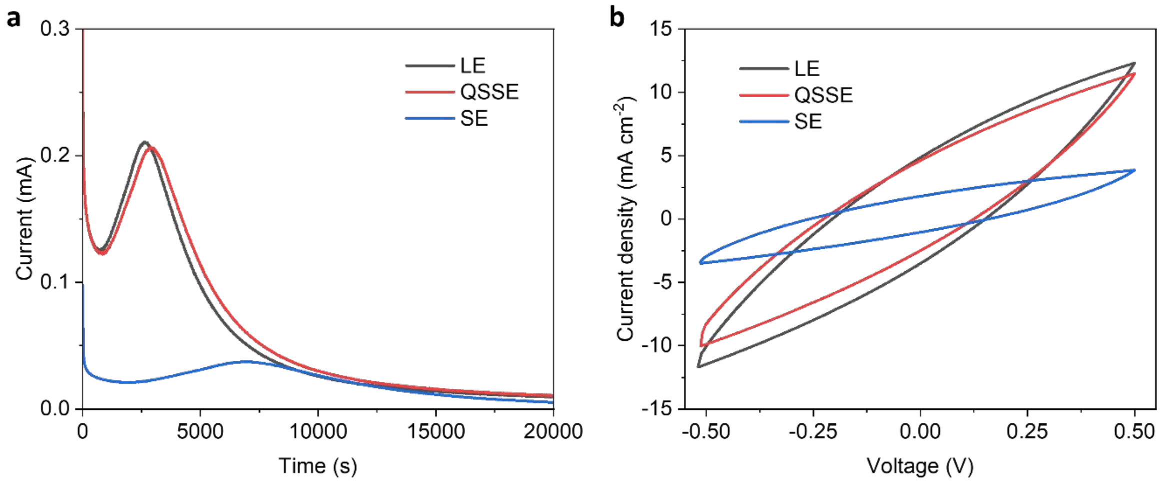

Figure S4. Electrochemical kinetics study of different electrolytes by Li_2S deposition and Li_2S_6 conversion measurements. **(a)** Potentiostatic discharge curves of Li_2S_8 solution at 2.05 V. **(b)** CV curves of Li_2S_6 symmetric cells at a scan rate of 50 mV s^{-1}.

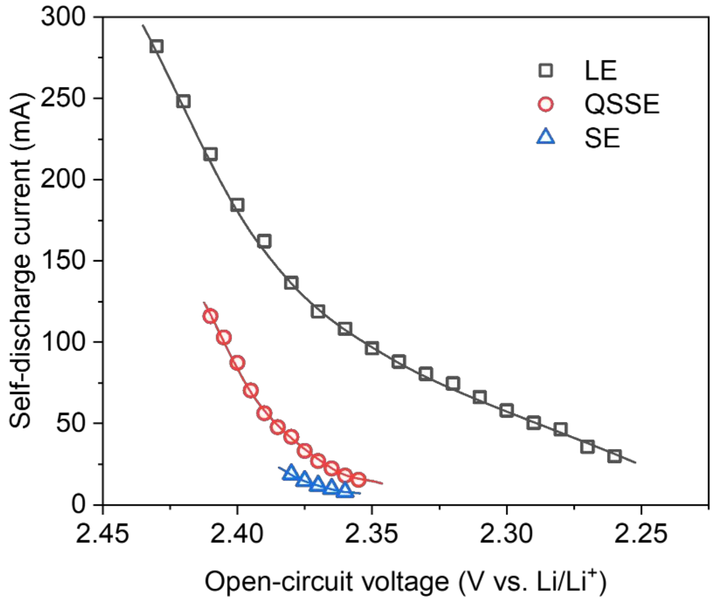

Figure S5. Self-discharge current of Li-S coin cells with different electrolytes. The solid lines indicate the evolution trend of self-discharge current over OCVs.

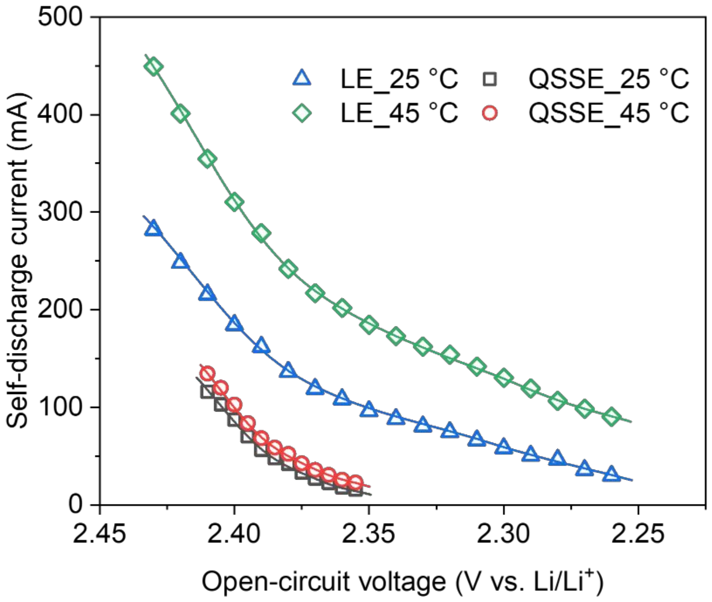

Figure S6. Self-discharge current of LE and QSSE-based cells with at various temperatures. The solid lines indicate the evolution trend of self-discharge current over OCVs.

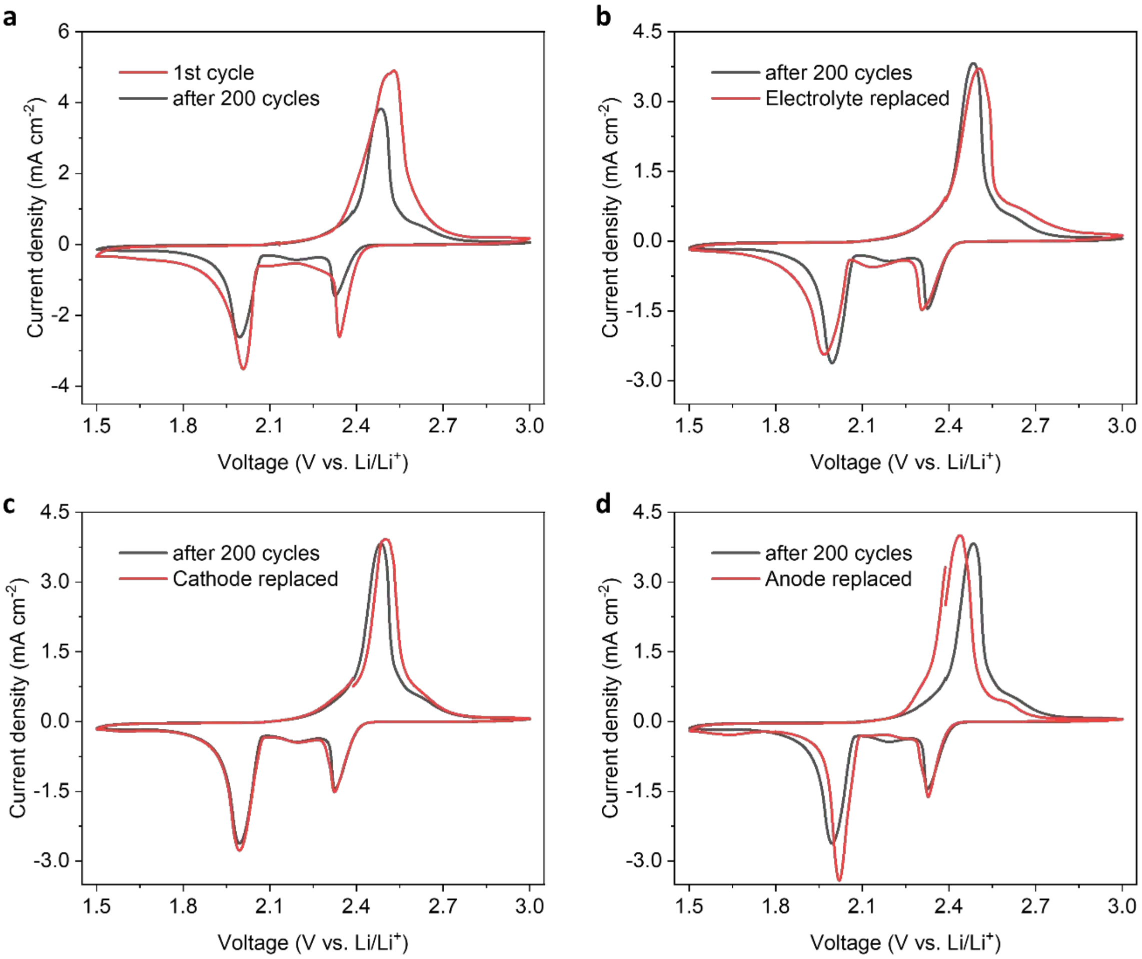

Figure S7. Battery degradation mechanism study by CV measurements for cells with different components replaced after 200 cycles. **(a-d)** Comparison of CV curves at a scan rate of 0.1 mV s^{-1} for the cell after 200 cycles with its initial cycle **(a)** and the 1st cycle after replacing the electrolyte **(b)**, cathode **(c)** and anode **(d)**.
